# A Comprehensive Review of Rollpave Pavement Technology: Current Research, Practices and Challenges

**DOI:** 10.3390/ma19061065

**Published:** 2026-03-11

**Authors:** Yanshun Jia, Mingyang Lan, Zeyu Wu, Haikun Lian, Chundi Si, Ying Gao, Shaoquan Wang, Linhao Gu, Zhuoran Li

**Affiliations:** 1 School of Traffic and Transportation, Shijiazhuang Tiedao University, Shijiazhuang 050043, China; jiaysh@stdu.edu.cn (Y.J.); 2202506003@student.stdu.edu.cn (Z.W.); 2 School of Civil Engineering, Shijiazhuang Tiedao University, Shijiazhuang 050043, China; 1202401106@student.stdu.edu.cn; 3 Hebei Shi-Tai Expressway Development Co., Ltd., Shijiazhuang 050090, China; hbst_jhhtb@163.com; 4 School of Transportation, Southeast University, Nanjing 211189, China; 230208342@seu.edu.cn; 5 College of Civil Engineering, Taiyuan University of Technology, Taiyuan 030024, China; wangshaoquan@tyut.edu.cn; 6 School of Civil Engineering and Architecture, Nanjing Institute of Technology, Nanjing 211167, China; gu_linhao@163.com

**Keywords:** rollpave pavement, rollable paving material, rollable asphalt mixture, evaluation criteria, paving construction technology, review

## Abstract

Rollpave technology offers an efficient and low-disruption solution for pavement rehabilitation but has not yet been widely implemented in practice. This review aims to provide a comprehensive overview of rollpave technology by examining performance evaluation methods, material design strategies, and construction workflows, and identifying its advantages and limitations to support practical application. Recent advances in rollpave pavement technology are reviewed, including flexural performance testing methods and evaluation criteria for rollable pavement materials, as well as the design of flexible asphalt mixtures and interlayer bonding materials. Construction techniques across different stages of rollpave implementation are summarized, and existing engineering case studies are reviewed. The advantages and limitations of rollpave technology are evaluated in comparison with other pavement construction and rehabilitation approaches, and current research focuses are discussed. The review indicates that pavement performance requirements can be achieved through the development of specialized modified asphalt binders and optimized mixture designs. On-site installation relies on coordinated operation of multiple devices to ensure adequate interfacial bonding between new and existing layers; however, current practices are largely experience-based and lack standardized guidelines. It is believed that rollpave technology demonstrates unique advantages for rapid pavement repair and emergency rehabilitation, but there are still challenges related to material and structural design, on-site installation, and cost-effectiveness that remain, limiting large-scale adoption. Future research could focus on establishing technical standards, developing specialized equipment, and enhancing multifunctional integration.

## 1. Introduction

The rapid expansion of the global road transportation network and the acceleration of urbanization in recent years have led to a sharp increase in the demand for road construction and maintenance. Traditional asphalt pavement construction and maintenance methods, characterized by long construction cycles, significant impact on traffic, and difficulty in controlling construction quality [1,2,3,4], are no longer adequate to meet the current demands for efficient and rapid road repairs. Especially in the context of accelerated urbanization, improving the efficiency of repair on heavily trafficked road segments and emergency roads has become an urgent issue in the transportation field. Designing pavement structural layers as convenient and detachable structures can significantly enhance the efficiency of rapid repair and maintenance operations. Against this background, rollpave pavement technology has emerged and gained widespread attention as a novel pavement surfacing solution.

**Figure 1:**
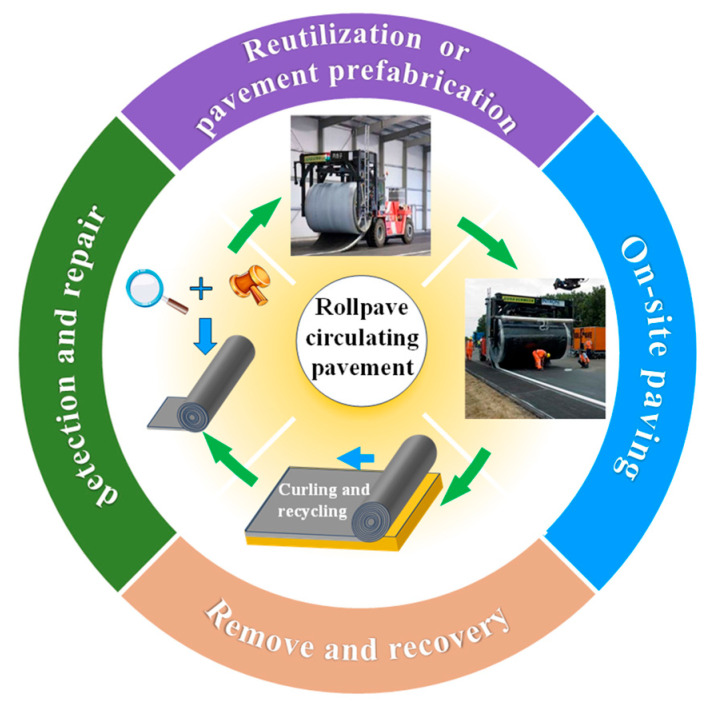
Logical concept diagram of prefabricated rollpave pavement.

Rollpave pavement originated from the “Road to the Future” project initiated by the Netherlands in 1996. In the Modular Road Surface technology of this project, the concept of rollpave pavement was proposed, and Dura VerMeer and Intorn companies developed the rollpave product and technology, which was named “rollpave” at that time [5,6]. Inspired by the flexibility of carpets, this concept aims to manufacture long pavement strips that can be wound into reels and unrolled during installation. Specifically, rollpave pavement is prefabricated in a factory, wound onto specialized reels, and transported to the construction site. During installation, the pavement is unrolled in a manner similar to carpet laying and rapidly bonded to the base layer via induction heating. When maintenance is required after its service life, the aged layer can be detached and replaced with a new prefabricated pavement. This technology significantly enhances transportability and operational flexibility. The complete process is illustrated in Figure 1.

**Figure 2:**
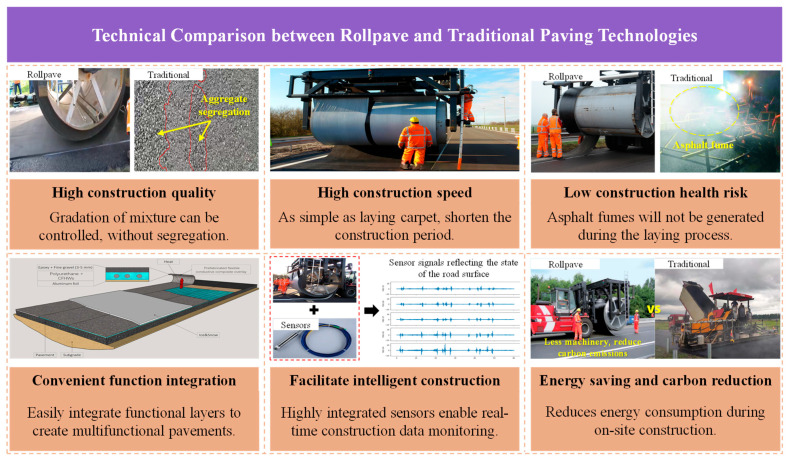
Comparison of rollpave and traditional paving technologies.

Compared to traditional pavement construction techniques, rollpave asphalt pavement technology offers several advantages. Regarding construction quality, traditional asphalt pavements are inherently susceptible to material performance variations (e.g., segregation) during the construction process, which often leads to non-uniform quality [3,4]. In contrast, rollpave technology ensures rigorous control over the asphalt mix design through factory prefabrication. Minimizing the performance variability during the transport and paving stages enhances overall construction quality. In terms of convenience, the on-site laying process is as straightforward as unrolling a carpet, which shortens construction and maintenance durations. This allows for the rapid reopening of traffic and alleviates the traffic congestion typically caused by maintenance activities [7,8,9]. Furthermore, the modular design of rollpave makes it suitable for temporary construction and emergency repairs, as it can be prefabricated in advance in the factory and its installation is less sensitive to climatic conditions, demonstrating exceptional flexibility and practical value. From the perspective of functional integration, the modular structure and prefabricated nature of rollpave facilitate the incorporation of specialized functional layers and embedded equipment, enabling the creation of multi-functional composite pavements. For instance, carbon fiber heating wires can be distributed within the mat to form a temperature-regulating overlay, which enhances electro-thermal conversion efficiency and effectively mitigates the impact of ice and snow on pavement structures and road safety [10]. Moreover, prefabrication allows for the seamless integration of internal sensing devices, facilitating real-time data collection and promoting intelligent pavement management. Finally, regarding environmental and health considerations, traditional paving generates substantial asphalt fumes. Rollpave technology reduces on-site energy consumption and carbon emissions by shifting material production to the factory. This approach decreases the release of hazardous substances and lowers health risks for workers, particularly in enclosed construction environments such as tunnels [11]. Based on the literature [5,9,10], a technical comparison between rollpave and traditional paving technologies was constructed, as illustrated in Figure 2.

Rollpave technology represents a paradigm shift in conventional asphalt pavement construction and maintenance, offering a promising trajectory for future infrastructure development [12,13]. However, despite its inherent advantages over traditional methods, the large-scale implementation of this technology faces substantial technical hurdles. Specifically, conventional pavement materials often fail to satisfy the more stringent performance requirements of rollpave structures. This is often accompanied by high upfront costs, which is one of the reasons why Dura Vermeer stopped expanding the application scale. Furthermore, the lack of standardized industrial specifications for the design, production, and construction phases has impeded the further evolution of rollpave. To address these gaps, the present study aims to provide a comprehensive review of the current state of rollpave materials and technologies. This review analyzed rollpave pavement materials, specifically flexible mixtures and interlayer materials, while identifying existing challenges. Additionally, construction techniques across different stages of the workflow were evaluated. Finally, several asphalt pavement construction techniques were compared, the limitations and challenges currently faced were analyzed, and insights and suggestions for future development were provided.

## 2. Methodology

In this study, a systematic literature review was conducted to identify the applications of rollpave technology in road engineering. First, considering that rollpave remains an underexplored yet potentially promising field in recent years, the overall objective of this study was defined as investigating and analyzing the current research status, practical applications, and potential challenges associated with rollpave technology in pavement engineering. Due to the limited number of available studies on rollpave pavements, relevant publications were manually collected from multiple academic databases one after another, including Web of Science, Engineering Village, Google Scholar, and China National Knowledge Infrastructure. Keyword searches were performed in each database. Keywords highly relevant to the topic include rollpave pavement, rollable paving material, rollable asphalt mixture and prefabricated carpet-covered flexible pavement. Other keywords used to assist in explanation include asphalt pavement, epoxy resin, interlayer, pavement performance, etc. Subsequently, existing studies on rollpave technology were reviewed from the perspectives of material design and construction processes, and both the resolved issues and the remaining technical challenges at each stage were discussed. Finally, based on the literature review and comparison with related pavement technologies, the advantages, limitations, and development potential of rollpave technology were analyzed, and possible reasons for its relatively slow advancement were explored.

**Figure 3:**
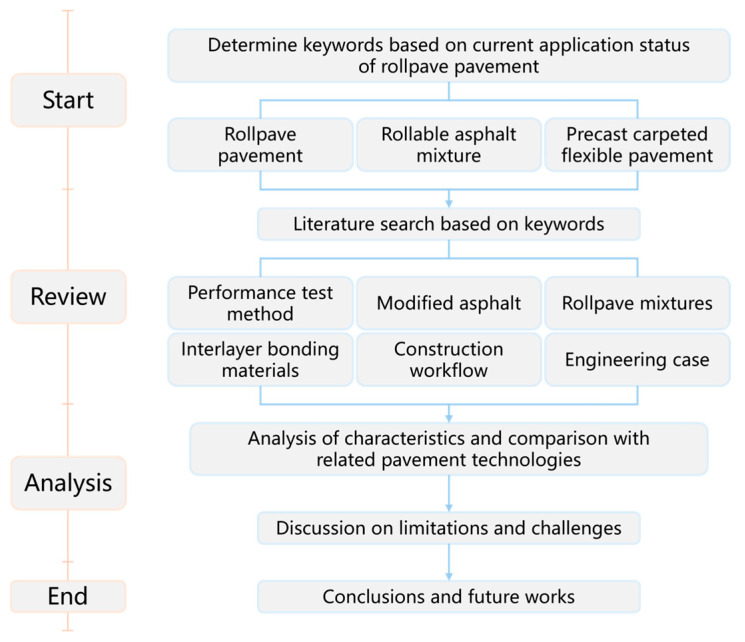
Structure of this review paper.

Through the literature retrieval and screening process, about 57% of the literature comes from Web of Science, about 9% comes from Engineering Village, about 15% comes from Google Scholar, and about 19% comes from China National Knowledge Infrastructure. Among them, fewer than twenty are related to material modification, development and performance research, about twenty are related to interlayer treatment and bonding performance, and about ten are related to construction technology, equipment, and quality control. In addition, 32 publications highly relevant to rollpave were further analyzed, including journal articles, conference papers, books, dissertations, and patents. Among these, eleven were sourced from Web of Science, five from Engineering Village, eight from Google Scholar, and eight from China National Knowledge Infrastructure. The publications span the period from 2003 to 2025: approximately 28% were published within the past five years, about 25% between five and ten years ago, approximately 28% between ten and fifteen years ago, and the remaining 19% more than fifteen years ago. These findings indicate that research in this field has progressed continuously but has remained relatively limited in scale, suggesting that rollpave technology possesses sustained development potential while possibly being constrained by certain technical, economic, or practical factors that have hindered rapid expansion. The overall structure of this review is illustrated in Figure 3.

## 3. Materials and Performance Evaluation of Rollpave Pavement

The excellent material property is a key factor driving the rapid development of rollpave pavement technology. Compared with traditional paving technology, the paving materials utilized in this technology require higher performance, including but not limited to commendable flexural strength [14]. This is to ensure the continuity and integrity of the entire structure during the curling process. Therefore, it is one of the main research interests related to rollpave pavement technology to optimize the composition of rollpave paving materials to improve the flexural performance. In addition, the quality of the pavement is directly influenced by the curling radius and thickness of the pavement; different combinations of curling radius and pavement thickness place different demands on the bending properties of the material [15]. The bending performance testing method that matches the process parameters is equally important for accurately evaluating and optimizing material properties.

### 3.1. Test Method and Evaluation Criteria for Flexural Performances of Rollpave Materials

**Figure 4:**
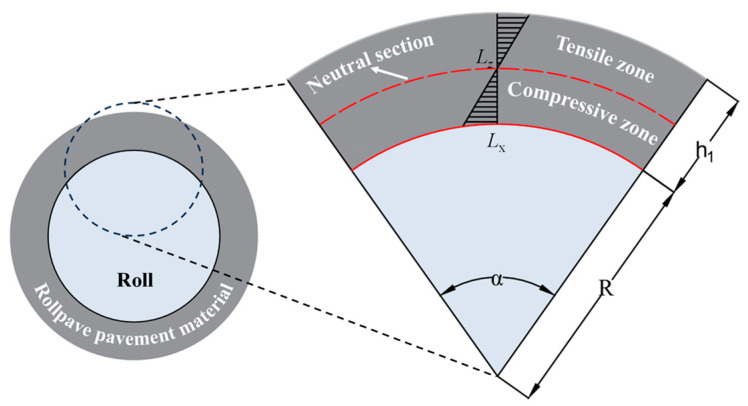
Force diagram during the curling process of rollpave pavement.

Coiling is a defining attribute of rollpave that ensures its distinctive convenience. This feature imposes specific requirements on the flexural performance of pavement materials. During the curling process, the surface of a rollpave pavement material at a roll is subjected to bending and tensile stress, and the bottom is subjected to compressive stress, as shown in Figure 4. To ensure that the rollpave mixture does not produce cracks that affect its strength during curling, the flexural performance should be given special consideration when designing the material. The flexural beam test is a common indoor test used to determine the flexural tensile properties and bending failure characteristics of materials. A previous study has shown that the distribution of stress on the mixture during the trabecular bending test is relatively close to that during the curling process [16]. At present, many researchers have used this test to evaluate the flexural performances of rollpave asphalt mixtures [15,17,18,19]. Assuming that the rollpave mixture is an isotropic homogeneous elastic body and the influence of gravity is disregarded, the bending performance index in this test can be calculated by the following Equations (1)–(3).

(1) L=α⋅R

(2) $$\varepsilon =\frac{L_{x}-L_{z}}{L_{z}}=\frac{\frac{h_{1}}{2}}{R+\frac{h_{1}}{2}}$$

(3) $$\varepsilon_{B}=\frac{6h_{2}d}{L^{2}}$$

where *L* is the span length of the specimen; *α* represents the radian of the central angle; *R* is the radius of the roll; *ε* denotes the approximate flexural-tensile strain at the bottom of the surface layer; *L_x_* and *L_z_* represent the arc length of the lower surface and the neutral surface of the surface layer; *h*_1_ is the thickness of the surface layer; *h*_2_ is the width of the mid-span section of the specimen; *d* denotes the mid-span deflection.

The above bending performance indexes can be used as criteria to judge whether an asphalt mixture can be used in rollpave pavement. Existing research [17,20] has assumed that in the trabecular bending test, there will not be cracks that affect its strength within the mixture during the curling process when the mid-span deflection of the beam specimen at failure is greater than the critical value calculated by the three equations. It can be inferred that this asphalt mixture can be used in rollpave pavement. Although the evaluation criteria and technical requirements for the bending performance of the mixture and the curling radius and pavement thickness were established by the bending test of a small beam, there are still limitations in the practical application of the rollpave pavement. On the one hand, from an experimental perspective, a significant discrepancy exists between the mechanical behavior observed in laboratory small-beam bending tests and the actual rolling/unrolling process. The non-linear and anisotropic characteristics of rollpave materials are frequently simplified in current studies, and the dynamic tension–compression cycles experienced during repeated coiling are difficult to fully simulate. In most current investigations, simplified loading scenarios are adopted, in which specimens are subjected to monotonic displacement-controlled loading under idealized simply supported boundary conditions. Such configurations cannot fully reproduce the complex stress state encountered during real curling and deployment. By contrast, during actual curvature, prefabricated mats undergo curvature-controlled deformation under continuous contact constraints, resulting in non-uniform stress gradients through the thickness and evolving into coupled tensile–compressive stress states along the curling path. These limitations hinder a comprehensive understanding of the material’s authentic mechanical response. Furthermore, small-scale laboratory specimens are insufficient to accurately represent the mechanical behavior of full-scale prefabricated mats under cyclic curling conditions, which further widens the gap between theoretical predictions and practical performance. Long-term on-site measurement data is needed to confirm the validity of laboratory results. On the other hand, from the standpoint of practical application, existing evaluation standards lack data regarding environmental factors and long-term performance. Specifically, the impacts of thermal cycling, moisture fluctuations, and prolonged storage on the bending properties and oxidative aging of rollpave materials remain under-researched, posing a risk of performance degradation prior to field installation.

### 3.2. Special Modified Asphalt for Rollpave Pavement

Rollpave pavement has higher requirements for the performance of asphalt materials. The asphalt materials used for rollpave pavement not only need to meet basic road performance, but also need to have good bendable ability, which is frequently not attained by ordinary asphalt materials. Consequently, there is a necessity for the asphalt modification techniques to enhance the road performance of asphalt binders. Adding various modifiers, such as polymers, to asphalt can improve the stress–strain relationship of asphalt and form a system with thermodynamic instability but dynamic stability, which has an important contribution to the improvement of the rutting resistance, flexibility and crack resistance of asphalt.

In recent years, a series of studies have carried out on the development of special modified asphalt for the special needs of rollpave pavement. Dong et al. [21,22] prepared a rollpave-specific modified asphalt using styrene-butadiene-styrene (SBS) particles, furfural-extracted oil, cycloalkane composite oil, plasticizer (C5 petroleum resin), compatibility stabilizer (sulfur), and self-developed rollpave-specific polymer particles based on ethylene–vinyl acetate (denoted as GTA). The microstructure analysis showed that with the increase in SBS and GTA content, the polymer continuous phase expanded significantly, and the asphalt dispersed phase decreased, forming a bicontinuous phase structure, which enhanced the cohesion and structural stability of the system. They also found that when the penetration is basically the same, the softening point of the special modified asphalt is about 2.21 times and 1.86 times higher than that of the matrix asphalt and SBS modified asphalt, and the ductility is 14.71 times and 2.59 times higher than that of the matrix asphalt and SBS modified asphalt respectively, which means mixtures using specialized modified asphalt may have better high-temperature stability and crack resistance. In order to further analyze the road performance of the special modified asphalt, the rheological test was carried out, and the results showed that the material has better high-temperature stability and low-temperature flexibility. They finally analyzed and proposed asphalt technical indicators suitable for rollpave pavement based on the test results.

Similarly, Shojaei et al. [23] proposed a rollpave modified bitumen (RMB) composed of ductile agents, thermoplastic elastomer (modified by ethylene propylene diene monomer) and bio-oil, whose performance indexes are as shown in Table 1. The softening point of the material is almost twice that of the virgin asphalt, and the ductility at low temperature is significantly improved, showing better toughness and brittleness resistance. Additionally, it was found that RMB has lower creep stiffness and good stress relaxation ability at low temperature, higher recovery rate and fatigue life at high temperature than that of virgin asphalt, showing excellent comprehensive rheological properties and durability.

As shown in Table 1, the test values for both types of special modified asphalts for rollpave pavement are similar, and both have high requirements for softening point and ductility at low temperature. However, performance test indicators still exhibit discrepancies across different studies. Therefore, it is still necessary to clarify which key performance characteristics of asphalt materials should be improved to fully meet the requirements of rollpave asphalt applications. Additionally, the performance evaluation index system for these materials requires further unification. In addition, the primary focus of existing research has been on enhancing high-temperature stability, low-temperature flexibility, and rheological properties, while relatively less attention has been paid to environmental factors, particularly the aging effects caused by ultraviolet radiation and their detrimental impact on pavement performance. Research indicates that ultraviolet radiation significantly accelerates asphalt aging, leading to reduced road performance and a shortened service life [24,25]. Severe gradient aging of asphalt can be caused by ultraviolet radiation, even when the penetration depth is relatively shallow [26]. In cases of thin film thickness, ultraviolet radiation even becomes the main cause of ageing [27]. Consequently, it is essential to undertake more methodical and comprehensive research on the ageing behaviors of rollpave pavement-specific asphalt materials in future studies. At the same time, the standardized performance evaluation method for special asphalt suitable for rollpave pavements has not been established, which limits the comparability of different research results and engineering applications.

### 3.3. Various Mixtures for Rollpave Pavement

The mixtures currently used for rollpave pavement include asphalt mixtures, cement-based mixtures, and epoxy resin-based mixtures. An increasing amount of research is being dedicated to optimizing these materials to meet the requirements of rollpave pavement in terms of bending performance and durability. This section aims to review the composition, properties, and optimization methods of the above-mentioned mixtures based on existing research.

#### 3.3.1. Asphalt Mixture for Rollpave Pavement

Traditional asphalt mixtures struggle to meet the requirements of flexural or durability performance for rollpave pavement [5,16,19]. To improve the bending performance and durability of materials, some researchers have optimized the composition design of asphalt mixtures. Dong [15] believed that the selection of mixture composition with reasonable volume parameters and excellent performance is an important guarantee for the performance of rollpave pavement. The gradation with excellent bending performance was determined by analyzing the influence of the passing percentage of the critical sieve on the bending performance. In addition, the high-temperature performance and bending performance of the mixture were also considered when determining the asphalt–aggregate ratio. Finally, Dong [15] successfully developed an asphalt mixture using a specially designed rollable modified asphalt binder for rollpave applications. Based on the working conditions of pavement thickness of 40 mm and curl diameter of 1.5 m, the corresponding evaluation standards for bending performance and road performance were established, which provided a reference for subsequent material optimization and engineering application. Similarly, Shojaei et al. [23] used the same grading design method to prepare an asphalt mixture containing a compounding agent (bio-oil additive) and a reactive agent, and analyzed its durability. They found that the asphalt mixture has strong flexibility, crack resistance, high-temperature performance and fatigue performance, which has potential for use as a prefabricated paving asphalt material.

**Figure 5:**
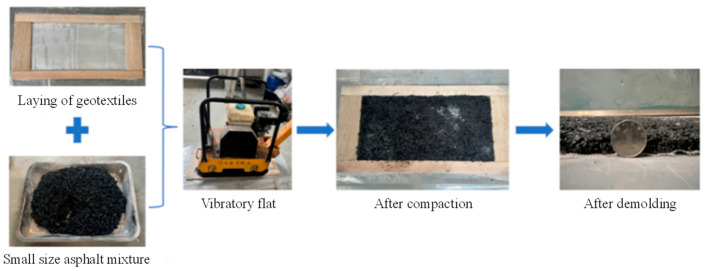
Preparation of rollpave pavement mixture with warp-knitted polyester glass fiber reinforced layer at the bottom [19].

**Figure 6:**
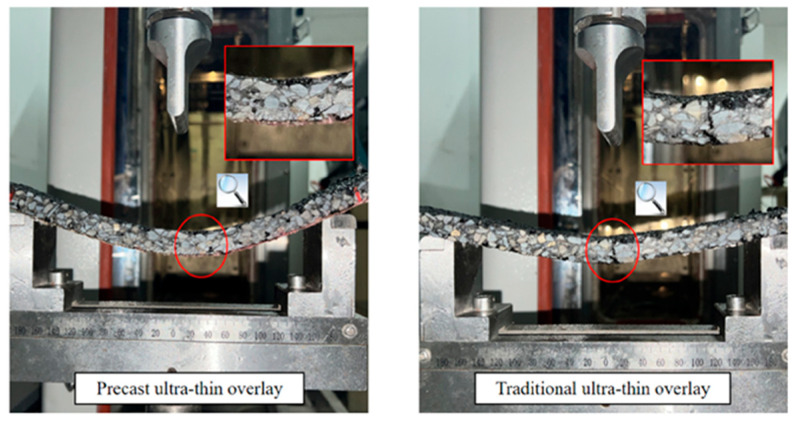
Comparison of the bending performance of the overlay before and after optimization [19].

In addition, based on the development of high-bending-performance asphalt mixtures, some researchers have further optimized the structure of rollpave pavements to improve their bending performance. For example, Dai [16] incorporated an emulsified asphalt binder layer of specified thickness at the bottom of the mixture, while Tan et al. [19,28] paved a warp-knitted polyester glass fiber reinforced layer at the bottom of the mixture, with the preparation process illustrated in Figure 5. This way of optimizing both the material and the structure can effectively address the shortcomings of the material in terms of its bending properties in rollpave pavements, achieving better curling ability than traditional ultra-thin overlay, as shown in Figure 6. However, the long-term durability of this kind of composite structure and its bonding performance with the original pavement still need to be studied further.

#### 3.3.2. Cement-Based Mixtures and Epoxy Resin-Based Mixtures for Rollpave Pavement

Apart from traditional asphalt mixtures, cement-based materials and epoxy resin materials are also used for rollpave pavements. On the one hand, as a common pavement material, cement-based material has the characteristics of high strength and high durability, but its stiffness is too high, its toughness is insufficient, and it is prone to cracking [29,30]. Given this situation, some researchers have successfully prepared a textile concrete with short fibers by combining polyvinyl alcohol fiber with glass fiber fabric grid to improve the bending properties of cement-based materials [31,32,33]. Although it has been demonstrated that adding fiber grids at the bottom can directly enhance performance, the extent to which the bending performance of textile concrete with short fibers meets the service standard of rollpave pavement, as well as its long-term performance and life-cycle cost, remains uncertain and requires further investigation. Meanwhile, most of the aforementioned studies rely more on “patch” reinforcement, which fails to solve the original problem of the mixture but instead increases project costs.

On the other hand, the epoxy resin-based materials, which have been confirmed to have excellent road performance and durability [34,35,36], can be widely used in bridge deck pavement, expressway maintenance, color pavement and special pavement [37,38]. However, epoxy resin has high crosslinking density, poor flexibility and is easy to crack [39]. Therefore, some researchers improve the bending properties of epoxy resin by adding some modifiers. Le [40] and Zhao et al. [41] developed a curable epoxy resin mixture (CERM) with epoxy resin, toughener (polyurethane), amine hardener, diluent and promoter. First, the optimal dosage of admixture was determined according to the deformation capacity and viscosity characteristics of the epoxy resin binder, and the mixture gradation was designed according to the technical specification [42]. Then, the optimal amount of epoxy resin binder in CERM was determined by bending performance and splitting strength tests. The mixture theoretically meets the bending performance requirements of 2.5 cm thickness and 1 m curling radius. In order to further improve the bending performance of the CERM, Le [40] applied a layer of polyester fiber cloth on the bottom of CERM, resulting in an approximate 15% increase in maximum deflection compared with the CERM. In general, while CERM shows excellent bending performance, its practical application, material cost, durability and other issues require further investigation. In addition, existing research lacks unified standards for temperature conditions, loading methods, and specimen dimensions in the performance testing of rollpave mixture. This results in poor comparability between evaluation results from different studies. Meanwhile, for these new rollpave pavement materials, the research on their long-term durability, fatigue performance, and damage evolution law under the coupling effect of complex environment (e.g., temperature and humidity changes, ultraviolet aging, and salt erosion) and traffic load is insufficient, which cannot provide a basis for their design life and reliability.

### 3.4. Interlayer Bonding Materials

The adhesive coating, a key functional layer within the pavement structure, plays a role in forming a continuous and reliable bonding interface between the extant pavement and the newly constructed pavement layer. This ensures that the interlayer is subjected to synergistic stress and possesses adequate bonding strength [43]. Under continuous vehicle loading, the interface shear stress may exceed the strength of the bonding layer, resulting in interlayer debonding [44,45]. This debonding can further lead to damages such as potholes and layer displacement, ultimately compromising the structural stability and service life of the pavement. In rollpave pavement, this phenomenon is more pronounced due to the inherent initial interlayer defects caused by its ultra-thin layer thickness and curled paving process. Therefore, it is crucial to choose suitable bonding coating materials to improve the interlayer bonding capability of rollpave pavement. Furthermore, the paving methods (e.g., hot paving and cold paving) significantly influence the interfacial bonding performance of rollpave. The selection of the appropriate method necessitates a comprehensive evaluation in conjunction with the material properties of the tack coat.

Currently, extensive research has been conducted to enhance the performance of these bonding materials. Table 2 summarizes the compositions and performance characteristics of interlayer bonding materials under various paving conditions as reported in the existing studies.

The summarized studies indicate that hot paving provides higher interlayer bonding strength and fatigue resistance, but with high energy consumption and construction difficulty. The cold paving method shows a relatively low bonding strength. Notably, waterborne epoxy resin emulsified asphalt demonstrates superior shear and tensile strength compared with SBS-modified asphalt and matrix asphalt. Among the reported materials, epoxy-based binders, especially waterborne epoxy resin emulsified asphalt, exhibit the best interlayer bonding performance. In summary, the interlayer bonding performance is governed by the synergistic effects of paving temperature, bonding material rheology and interface structural compatibility.

Despite the fact that researchers have comprehensively investigated bonding layer materials and examined their strength characteristics and construction adaptability, systematic comparative analyses tailored to the specific application scenarios of rollpave pavements remain inadequate. Existing conclusions still require further refinement in terms of their engineering relevance and the comparability of research outcomes. The application of hot paving bonding layer materials (e.g., SBS modified asphalt) requires heating processes, which leads to higher energy consumption and relatively complex construction techniques. However, these materials offer superior bonding properties. This increases the overall installation complexity of rollpave pavement. In contrast, cold paving materials like emulsified asphalt and certain modified emulsified asphalts offer significant advantages in construction convenience. However, their bonding strength decreases in low-temperature environments, leading to insufficient interlayer adhesion. This can subsequently induce pavement defects such as cracking and interlayer delamination.

## 4. Construction Workflow and Performance Determinants of Rollpave Pavement

The rollpave technology enables rapid deployment of pavement structures [14] and is categorized as a form of prefabricated pavement construction. As illustrated in Figure 1, the process begins with the factory-based prefabrication of the surfacing layer, which is subsequently hoisted and transported to the construction site for direct installation onto the milled existing pavement structure. This integrated approach, combining factory prefabrication with on-site laying, effectively mitigates the inherent drawbacks of traditional paving, such as imprecise fabrication, operational inefficiency, and challenges in quality control. Furthermore, it facilitates standardized construction and refined management practices for pavement engineering [11,63]. This section outlines the primary phases of rollpave construction and evaluates the potential impacts of each stage on the resulting pavement performance.

### 4.1. Road Milling and Planing

Rollpave pavements are primarily applied in rehabilitation projects of heavily trafficked roads, where the existing surface layer is milled off while the structurally sound base or binder course is retained. A bonding layer is then applied before installing the prefabricated asphalt mat, enabling rapid construction and early traffic reopening without disturbing the subgrade. The thickness of rollpave pavement is fixed and cannot be changed; it is required that the original pavement foundation has a higher level of smoothness and texture roughness to ensure a flat road surface after laying rollpave pavement. Road milling is the use of a milling machine to quickly and efficiently remove the upper layer of the old road surface, in order to provide a smooth and clean base for the newly laid rollpave-style asphalt pavement. Milling operations can be divided into standard milling, and micro milling based on the density of tool spacing on the milling rotor [64]. The pavement milling process is particularly suitable for high-traffic highways and urban roads, and can quickly and effectively deal with pavement damage problems [65,66], which coincides with the advantages of rollpave pavement.

Milling parameters (e.g., cutter spacing and milling speed) influence multiple aspects of pavement construction performance. Cutter spacing directly affects the resulting surface texture characteristics, including the mean texture depth and the ridge-to-valley depth (RVD). Higher milling precision generally leads to smaller RVD values; for example, the RVD of conventional milling is typically around 8 mm, whereas that of micro-milling can be reduced to approximately 3–4 mm [67]. Clearly, the post-milling surface roughness is closely associated with the required dosage of the relatively expensive bonding agents used in rollpave technology. If the RVD is large, a greater amount of bonding material is theoretically needed to fill the macroscopic texture generated by milling. Meanwhile, an overly thick bonding layer may induce shear flow at high temperatures, thereby increasing the risk of rutting. Milling operations also affect pavement functional performance. For instance, Gao et al. [64] have reported positive correlations between milling precision and milling speed with skid resistance. In addition, unlike rigid precast concrete slabs, rollpave pavements are composed of flexible asphalt-based materials. Local depressions left by milling, if not completely filled, may form micro-cavities. Under traffic loading and hydrodynamic pressure, these defects can readily lead to early distress or functional failure of rollpave pavements. Therefore, precise control of milling depth is essential.

Overall, milling plays a critical role in pavement construction, and milling quality directly determines the performance and durability of the subsequent pavement layer. In recent years, milling technology has made progress in equipment diversification, high-precision control, automation, and intelligence. For example, in order to obtain greater pavement roughness and texture quality, it is necessary to develop high-level milling rotors. Zuo et al. [68] explored and designed an advanced milling rotor and driving control system, enabling the accuracy and efficiency of milling operations. Meng et al. [69] developed a supervised Hebbian learning single neuron adaptive PID controller for the power control of cold milling machines, which meets the needs of adaptive power control for cold milling and planning equipment. Zuo [70] examined a digital milling system composed of the RD-M1 and RD-MC modules, achieving millimeter-level adjustment accuracy of milling depth. Liu et al. [71] proposed power adaptive control and limit load control to solve the problem of high energy consumption in milling machines, taking into account the characteristics of milling operations and cutting force models. In summary, through the improvement of the milling rotor and equipment control system, milling technology can better meet the requirements of smoothness and texture roughness of pavement base, and provide a potential guarantee for the smooth implementation of rollpave pavement.

### 4.2. Factory Prefabrication Process

The factory prefabrication process of rollpave pavement represents a fundamental departure from conventional in situ asphalt paving practices, adopting instead an industrialized production line that ensures high quality, uniform structural properties, and rapid on-site deployment. The prefabrication procedure can be divided into three principal stages:

(a) Material preparation and mix design: Specially modified asphalt binders and aggregates are selected to satisfy the dual requirements of high deformability during curling and sufficient stiffness under service loads. Previous studies have demonstrated that the incorporation of ductile modifiers (e.g., SBS polymers and rubber particles), together with carefully optimized aggregate gradations, can significantly enhance bending strain capacity while maintaining adequate mechanical resilience [22].
(b) Layer formation and compaction control: Under controlled factory conditions, the asphalt mixture is laid onto a continuous moving belt or precast mold system and subjected to calibrated compaction, vibration, and shaping procedures. During this stage, key parameters (e.g., temperature, compaction energy, and layer thickness) are strictly regulated to ensure structural homogeneity and to prevent internal defects such as segregation and excessive air voids, which could adversely affect both rollability and long-term pavement durability.
(c) Cooling, curling, and storage: After achieving the target compaction level and thermal stability, the prefabricated mat is gradually cooled. Once sufficient stiffness is attained, the continuous mat is wound onto drums or reels to form discrete rollpave units. These units are subsequently labeled, stored, and transported to construction sites for installation. Quality assurance at this stage includes verification of geometric dimensions, density uniformity, and bending performance to ensure consistent in-service behavior.

**Figure 7:**
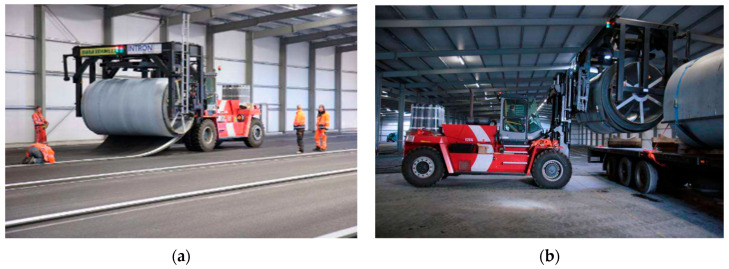
Prefabrication process of rollpave pavement [11]: (**a**) The process of curling rollpave pavement; (**b**) the rollpave pavement ready for transport.

The main prefabrication process in the factory is shown in Figure 7.

In terms of practical prefabrication implementation, Dong et al. [15] proposed a rollpave prefabrication process based on controlled paving speed, high-temperature compaction, and low-speed curling, providing a feasible industrial manufacturing route. Based on the above prefabrication process, two interrelated technical challenges can be identified. First, the asphalt mixture must achieve an optimal balance between flexibility and stiffness, allowing the mat to be rolled without cracking while providing adequate resistance to load-induced deformation during service. Previous material studies have shown that appropriate modifier selection and gradation optimization can significantly improve bending performance and low-temperature strain capacity, which are critical for rollpave pavement applications [11]. Second, unlike conventional hot-mix asphalt paving where construction parameters can be adjusted in real time, prefabricated mats must achieve strict dimensional and mechanical consistency prior to leaving the factory. Any variation in thickness, density, or binder content may lead to performance variability and increase the risk of localized premature failure. Therefore, laboratory development and pilot-scale production must be supported by rigorous and systematic process control frameworks.

### 4.3. On-Site Paving Process

Following the fabrication of the reel with rollpave-style pavement, the ensuing step is its installation on the road. The following steps should be taken to ensure a successful installation process: (1) Clean the original pavement base to completely eliminate floating, sinking, soil, debris, and moisture; (2) install the reel with rollpave-style pavement transported to the site onto the paving equipment; (3) spray adhesive coating evenly on the original pavement base and wait for the adhesive coating to form initial strength; (4) run the paving device and align one end of the rollpave pavement with the required splicing surface layer before paving; (5) to ensure good contact between the rollpave pavement and the original pavement base, a roller is used for compaction; (6) pour the joint material into the joint between the rollpave pavement and the required splicing surface layer.

**Figure 8:**
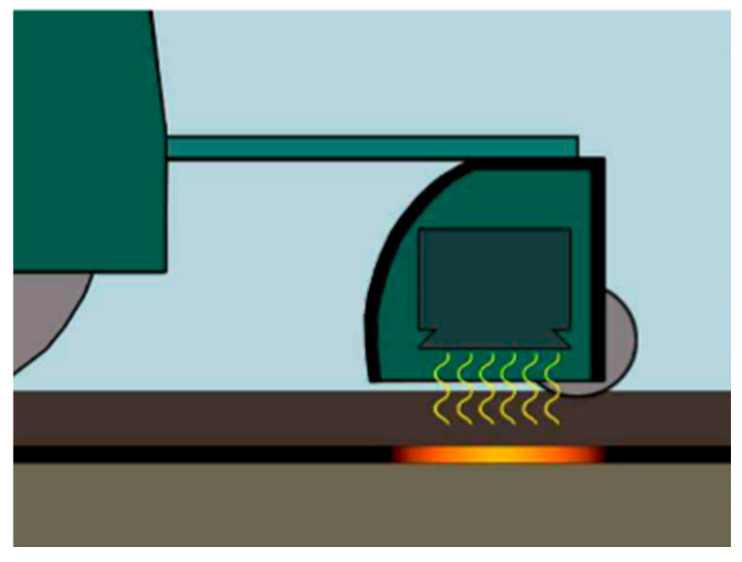
Heating the rollpave asphalt mixtures with electromagnetic induction equipment [11].

To achieve rapid bonding between the original pavement and the rollpave pavement, researchers [5] chose to mix metal materials into the bottom bonding layer of the rollpave mixture and use electromagnetic induction heating for efficient construction, as shown in Figure 8. An uneven distribution of metal materials may cause local overheating of the asphalt mixture, weakening the overall mechanical properties and durability of the bonding layer and asphalt mixture [72,73,74,75], which is difficult to meet the requirements for long-term use.

Additionally, to validate the applicability of rollpave asphalt pavement construction under low-temperature conditions, Dong [15] conducted experimental research on rollpave asphalt paving at temperatures ranging from 1 to 3 °C. During construction, tracks were laid on both sides of the pre-paved section. Rollpave pavement rolls were transported to the site by forklift and gradually unrolled at a speed of 1–3 m/min. To ensure the quality of the paving, gas torches were used to heat the underside of the rollpave pavement and the tack coat (SBS-modified hot mix asphalt) during construction. After completion of the paving, two compaction passes were performed. Furthermore, heated modified asphalt grout was used to seal the joints between the rollpave pavement and the existing pavement.

In summary, the construction workflow of rollpave has been systematically reviewed. Milling establishes the foundation for successful paving, factory prefabrication represents the core component of rollpave technology, and on-site installation ensures the interfacial bonding between the new and existing layers, ultimately realizing the technology’s objectives. However, at the current stage of development, rollpave construction technology faces several critical challenges. Specifically, there is a lack of research regarding the correlation between milling techniques and rollpave performance, and specialized technical indicators remain poorly defined. Critical parameters (e.g., milling speed, paving speed, compaction temperature, rolling passes, and layer thickness) are currently determined based on conventional specifications or empirical knowledge. The precise impacts of these parameters on precast quality, potential damage during compaction, and long-term performance remain unclear, which limits the stability and repeatability of construction quality. Finally, achieving uniform temperature distribution during induction or manual heating during installation proves challenging. This usually leads to localized overheating, which adversely affects the mechanical properties and durability of both the bonding layer and the asphalt mixture.

### 4.4. Engineering Case

**Figure 9:**
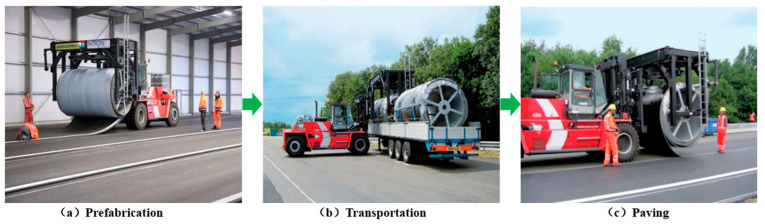
Construction process of the Netherlands’ rollpave pavement.

**Figure 10:**
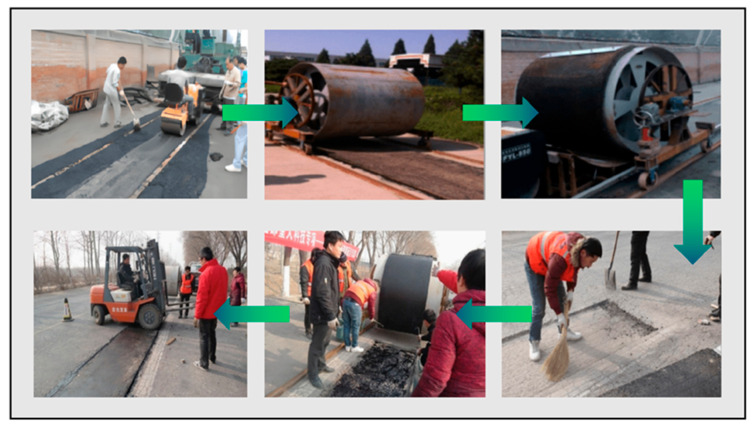
Construction process of rollpave pavement [11].

Presently, the practical application of rollpave pavement remains relatively limited, with existing implementation cases primarily concentrated in the Netherlands and China. Based on existing investigations [11,76], the Netherlands has constructed seven test sections of rollpave asphalt pavement, with a specific paving process shown in Figure 9. Research on rollpave pavement in China began relatively late but has already achieved certain progress. Researchers conducted a trial installation of rollpave asphalt pavement in Tongzhou District, Beijing [17], with the paving process illustrated in Figure 10. The performance of this test section in Tongzhou meets the requirements of road specification and is applicable to various highway and urban road scenarios. After consulting, the relevant information regarding rollpave pavement sections that have been implemented in different regions is presented in Table 3.

Regarding the materials used in these engineering cases, in addition to asphalt-based binders, synthetic alternatives such as polyurethane and epoxy resins have also been employed [7]. However, due to their high research and development costs and stringent technical requirements during construction, these novel materials remain at the experimental stage and have not yet been implemented on actual test pavements [77].

In summary, rollpave pavement technology has accumulated substantial engineering experience in the Netherlands and China, demonstrating its fundamental feasibility. However, this technology is still in the theoretical research stage; its practical application remains limited globally. Future efforts should focus on advancing engineering demonstrations of this technology across diverse geographical and environmental conditions. Such demonstrations would provide practical evidence to support standardization and large-scale implementation.

## 5. Discussion on Characteristics and Limitations of Rollpave Technology

### 5.1. Comparative Advantages and Application Potential of Rollpave Pavement Technology

Owing to the inherent advantages of prefabricated and assembly-based pavement technologies, prefabricated pavements have attracted increasing attention in the field of road engineering in recent years. At present, research on prefabricated asphalt pavements, both domestically and internationally, mainly focuses on prefabricated asphalt pavement slabs and rollpave pavement technology. By overcoming the shortcomings of traditional in situ pavement construction, such as extensive production processes, low construction efficiency, and difficulties in quality control, prefabricated pavement technologies enable standardized structural construction and refined management of pavement systems. In addition, thin overlay technology has gradually become one of the research hotspots in the field of preventive pavement maintenance due to its significant performance improvement effects and high construction efficiency. Taking conventional pavement construction as a reference, Table 4 compares prefabricated asphalt pavements, rollpave pavements, and thin overlay technologies in terms of their key characteristics, aiming to analyze the differences among these techniques and to clarify their respective applicability.

It can be observed that various pavement rehabilitation and maintenance technologies aim to shorten on-site construction time and reduce traffic disruption. Specifically, thin overlay technologies are primarily applied for functional restoration, such as improving skid resistance and surface durability. They are most suitable for preventive maintenance of pavements with sound structural conditions rather than for structural rehabilitation, with the main objective of extending pavement service life. Although thin overlays exhibit high construction efficiency and favorable cost-effectiveness, they are not suitable for repairing severe structural distress or for large-scale emergency pavement rehabilitation. In addition, thin overlay technologies are relatively sensitive to environmental conditions, and their forming quality may be adversely affected under low-temperature or high-humidity conditions.

In contrast, the main driving force behind prefabricated pavement technologies, including prefabricated asphalt pavement slabs and rollpave pavement systems, lies in the potential for substantially accelerated on-site installation. Traditional asphalt pavement construction typically involves transportation of hot materials, on-site mixing, paving, and compaction, which often results in prolonged lane closures. Prefabricated pavement technologies transform road construction into an assembly-oriented process, in which most operations (i.e., mixing, paving, and compaction) are shifted from the construction site to factory-controlled environments, while on-site activities are largely limited to the installation of finished pavement components. This approach not only significantly shortens construction duration—for example, rollpave systems have been reported to complete highway resurfacing approximately 50% faster than conventional methods [77]—and reduces road closure times (prefabricated asphalt slabs are often ready for traffic immediately or within minutes after placement [11]), but also mitigates environmental impacts. Conventional asphalt pavement construction is energy-intensive, and uncontrolled emissions of hazardous fumes and greenhouse gases are commonly generated by mixing equipment, diesel-powered machinery, and heating devices during on-site paving, particularly during the mixing stage [78]. In prefabricated systems, asphalt mixing and paving are conducted in factories, where cleaner energy sources (e.g., natural gas or electric heating) and fume-capture systems can be employed, enabling more effective emission control. Consequently, on-site toxic emissions are significantly reduced, lowering environmental pollution and occupational health risks [11]. Furthermore, factory prefabrication enhances functional integration of pavements. Road markings can be applied in advance before delivery, and embedded technologies such as sensors and heating coils can be pre-installed within prefabricated modules [79]. Factory-based production also reduces dependence on on-site weather and temperature conditions, allowing pavement maintenance operations to be conducted during cold seasons or light rainfall. Nevertheless, these technologies are associated with relatively high initial costs and a more complex construction organization.

Although prefabricated asphalt pavement slabs and rollpave pavements share many similarities, rollpave technology offers several distinct advantages. For prefabricated asphalt slabs, panel dimensions are constrained by handling, transportation, and installation requirements, with maximum slab sizes typically limited to approximately 1.5 m × 1.0 m [13], making them unsuitable for large-area continuous mainline paving. Smaller slab sizes lead to a higher number of joints, which is detrimental to pavement continuity, whereas larger slabs introduce logistical challenges during transportation. By contrast, the inherent rollability of rollpave pavements enables the transportation of larger continuous pavement units. Combined with their adaptability to environmental and climatic conditions, rollpave systems exhibit significant potential for large-scale emergency pavement rehabilitation scenarios, such as those caused by floods or earthquakes. In addition, the electromagnetic induction bonding technique employed in rollpave systems allows the bond between the pavement layer and the base to be reversible. When removal or replacement is required, electromagnetic induction can be used to release the pavement layer [3], facilitating long-term material recycling and sustainable development. Additionally, rollpave paving can also serve as a carrier for intelligent road surfaces, enabling the collection of traffic data such as vehicle speed, flow rate, ground pressure, temperature, humidity, and road rain and snow conditions [80]. Overall, the selection of appropriate pavement construction methods should comprehensively consider structural conditions, time constraints, traffic impacts, and economic performance. Given its unique advantages, rollpave pavement technology warrants further development and promotion in future applications.

### 5.2. Limitations and Challenges

Despite its considerable potential in future pavement construction, rollpave technology faces several limitations and challenges related to structural performance, on-site installation, and cost-effectiveness, which currently restrict its large-scale implementation. The major bottlenecks are summarized as follows.

In the ordinary way, most conventional pavement materials do not inherently satisfy the specific performance requirements of rollpave pavements. Structurally, the mechanical behavior of rollpave pavements differs from that of cast-in-place asphalt. In addition to traffic loading, rollpave pavements must withstand stresses induced during fabrication, transportation, and installation (e.g., bending during rolling and unrolling), resulting in tensile stresses at the bottom, compressive stresses at the top, and the inevitable formation of microcracks [11,17]. To enable prefabricated rollpave asphalt pavements to perform similarly to conventional pavements, researchers have addressed this issue through experimental design and optimization of mixture formulations with enhanced flexural resistance. However, asphalt mixture performance is influenced by design parameters such as pavement thickness and bending radius, and the relationships among these factors remain largely theoretical. To date, most curling performance evaluations rely on small-beam bending tests as substitutes, while a systematic material performance evaluation framework and factory production standards have yet to be established. Unified core performance indicators and recommended testing conditions are still lacking. Meanwhile, rollpave pavement technology offers flexibility in material selection, and alternative binders, such as epoxy resin, polyurethane, and resin-modified mixtures, have been explored and applied in rollpave pavement design. However, their high costs have thus far limited widespread application [11].

Regarding on-site installation, large-scale pilot projects in the Netherlands have demonstrated that, using rollpave technology, a 450 m section of highway can be closed overnight or during weekends, resurfaced, and reopened to traffic within a short period, confirming the feasibility of rapidly installing hundreds of meters of pavement within hours rather than days [5]. Nevertheless, studies indicate that certain operations, such as alignment and deployment of heavy pavement rolls, still require improvement to further enhance installation speed. Advanced roll-deployment equipment and improved bonding techniques remain subjects for future development, and standardized customized machinery and construction procedures have yet to be established. Another critical concern involves the behavior of prefabricated pavements at joints and their interaction with the underlying base. Adhesive materials are required to fill the joints between adjacent pavement panels as well as the interfaces between new and existing pavement layers. Appropriate milling operations and joint treatment are therefore critical to maintaining structural continuity and preventing premature distress or failure. There are also successful trials that have employed high-performance adhesives and innovative bonding techniques to ensure continuity between pavement layers. For example, existing study has used electromagnetic induction to achieve the uniform and reliable bonding between layers, without the need for a conventional tack coat [5]. Although field evaluations to date indicate that properly installed prefabricated asphalt pavements can carry traffic smoothly without severe damage during the observation period [5,17], installation methods vary among studies. Unified paving procedures and standardized construction parameters have not yet been established, and the limited availability of long-term performance data further constrains practical engineering validation.

Regarding the standardization of techniques and processes, at present, mixture design methods, flexural performance evaluation criteria, specimen dimensions, loading conditions, and construction parameters vary among different studies and pilot projects. Such inconsistencies not only limit cross-study comparability but also hinder the establishment of reliable and widely applicable performance benchmarks and safety margins. Without standardized design frameworks and technical specifications, it is difficult for industry stakeholders and transportation agencies to assess structural reliability, predict long-term performance, or develop quality control strategies suitable for industrial production. Consequently, the lack of codified guidelines represents a critical obstacle to the transition of rollpave technology from experimental validation to systematic engineering application. Establishing unified material design systems, performance-based evaluation criteria, and construction standards is therefore one of the prerequisites for its broader adoption.

From an economic perspective, the rollpave system seems to involve higher upfront costs than conventional paving methods. These additional expenses arise from research and development of specialized materials (e.g., polymer-modified binders and bonding agents), prefabricated processes in a factory, transportation of large prefabricated units, and the uncoordinated construction caused by equipment that has not yet been standardized. However, life-cycle economic performance presents a more nuanced picture. Tayabji et al. [81], using a life-cycle cost analysis (LCCA) model, reported that although material and transportation costs of prefabricated technologies on busy urban highways were approximately 20–50% higher, reductions in user delay costs due to significantly shortened construction windows led to an overall LCCA reduction exceeding 30%. These findings suggest that accelerated construction and minimized road closure durations may substantially offset higher initial investments, particularly on high-traffic corridors. Additional potential economic benefits include reduced on-site labor demand, lower traffic management costs, and the possibility of targeted modular repairs that avoid unnecessary replacement of structurally sound pavement areas. Moreover, the ability to install prefabricated pavements under less favorable weather conditions may further reduce the risks of schedule delays. Nevertheless, the economic considerations mentioned above still largely remain at the theoretical level, and the lack of long-term field performance data makes comprehensive LCCA of rollpave pavement technology challenging, making it temporarily difficult to confirm the claimed advantages. Achieving an optimal balance between performance benefits and cost-effectiveness, therefore, remains one of the key challenges for the broader adoption of rollpave pavement systems.

## 6. Conclusions and Future Works

### 6.1. Conclusions

This paper reviewed the latest research progress on rollpave pavement technology. After introducing and analyzing the current development of bending performance testing methods, performance evaluation criteria, material design, and paving technologies for rollpave pavements, as well as summarizing their advantages and limitations, the following conclusions can be drawn:

(1) Conventional pavement materials based on base asphalt or modified asphalt generally struggle to simultaneously satisfy the rollability and in-service performance requirements of rollpave pavements. To avoid crack formation during prefabrication and deployment, flexural strength, ductility, and rheological properties of asphalt mixtures have become key research focuses. Rollpave technology offers flexibility in material selection, and modified materials such as epoxy resin, polyurethane, and crumb rubber are potential options. Researchers have developed prefabricated asphalt mixtures by designing specialized modified binders and optimizing mixture compositions, demonstrating excellent high-temperature rutting resistance, low-temperature cracking resistance, and moisture stability, sometimes outperforming unmodified and SBS-modified asphalt. The flexural performance of rollpave pavements can be further enhanced by incorporating emulsified asphalt layers or polyester fiber fabrics. These performance advantages indicate that the structural performance gap between factory production and on-site construction can be bridged through advanced mixture and bonding material design.
(2) Compared with conventional hot-mix paving, rollpave pavements offer the potential for significantly accelerated on-site installation. Previous studies have demonstrated the feasibility of rapidly installing several hundred meters of pavement within hours, and construction efficiency is expected to further improve with the optimization of equipment and processes. Rollpave technology provides better controllability of production quality, reduces road closures and traffic disruption, facilitates functional integration, and aligns well with sustainability and environmental protection objectives. Nevertheless, disadvantages such as higher initial costs and more complex construction organization remain.
(3) Unlike conventional asphalt pavements, rollpave pavements undergo rolling and unrolling during prefabrication and on-site deployment, which induces tensile stresses at the bottom and compressive stresses at the top of the pavement layer. To prevent fracture of prefabricated pavements, it is necessary to analyze the rolling process and flexural performance. To date, no dedicated testing method has been established specifically for evaluating the flexural performance of rollpave pavements, and small-beam bending tests are commonly used as substitutes. Evaluation criteria based on mid-span deflection and bending strength/strain have been proposed to determine whether a material is “rollable.”
(4) Due to their inherent characteristics, rollpave pavements are more susceptible to interlayer debonding and therefore require particular attention. Epoxy-based adhesives have generally demonstrated superior interlayer bonding performance, while the actual bonding effectiveness is also influenced by paving temperature and interfacial material compatibility. In addition, rollpave pilot sections have successfully employed bonding techniques based on electromagnetic induction heating to ensure interlayer continuity, which can also be used to facilitate the removal of existing rollpave pavement, thereby supporting low-carbon recycling and promoting material circularity.
(5) Although rollpave technology shows considerable promise for future pavement construction, it remains at a relatively early, predominantly experimental stage, and large-scale implementation is still some distance away. Despite existing limitations and challenges in structural performance, on-site installation, and cost-effectiveness, rollpave pavements offer combined advantages, including large-area deployability, strong adaptability to climatic and environmental conditions, and convenient installation and removal. These characteristics give rollpave technology unique potential for large-scale emergency pavement rehabilitation, warranting continued research and development efforts.

### 6.2. Future Works

Research on rollpave asphalt pavements is still at an early stage. To transition rollpave technology from experimental studies to large-scale industrial application, future research should focus on the following strategic directions:

(1) Development of realistic cyclic rolling test methods: The evaluation of the bending performance of existing rollpave pavement materials is mainly based on single loading tests, which is difficult to reflect the characteristics of repeated loading under cyclic rolling conditions. Future research should focus on developing a special loading device that can simulate the repeated rolling and unrolling cycle within the constraints of the curling radius. It should also establish an evaluation system in which the core indicators are the number of cycles, strain and deformation.
(2) Specialized equipment development and intelligent integration: Rollpave construction relies on coordinated operation of multiple devices, and insufficient coordination between equipment functions and construction processes can compromise efficiency and stability. Dedicated transportation and paving equipment specifically designed for rollpave technology should be developed. Integrated machinery combining high-precision milling, automatic roll deployment, and uniform induction heating may be a promising direction. Furthermore, leveraging the prefabricated nature of rollpave pavements, future studies could explore embedding sensing devices and energy-harvesting modules (e.g., carbon-fiber heating elements) directly into factory production lines. This would enable rollpave pavements to serve as carriers for intelligent transportation systems, supporting real-time structural health monitoring and active deicing. Integration of rollpave technology into digital construction workflows or its combination with automated construction equipment is also recommended.
(3) Establishment of unified standards and specifications: In future, the quantitative relationship between the curl radius and the bending performance index of rollpave mixture should be clarified. The specimen size, loading conditions and evaluation threshold should also be uniform.
(4) Climate- and environment-oriented performance design: Pavement design tailored to specific climatic or environmental conditions should be considered. Coupled multi-factor analyses involving ultraviolet radiation, salt erosion, and thermal cycling are recommended to study the softening point, ductility and rheological performance of rollpave pavement materials, enhance climate adaptability and improve the resilience of pavements to damage caused by extreme events, thereby strengthening transportation infrastructure resilience. Further research may exploit the modular nature of rollpave pavements to enhance functional integration. For example, pavement designs could incorporate drainage and noise-reduction features, exhaust-emission mitigation functions, as well as embedded devices such as monitoring sensors and energy storage systems, thereby supporting the development of future smart road infrastructure.
(5) Expansion of field applications and long-term monitoring: Increasing engineering applications and maintaining long-term performance monitoring are essential. The stability, flatness, joint integrity, and structural damage evolution of the pavement should be closely monitored. This can verify long-term on-site performance and provide data for LCCA. Future research should focus on balancing performance benefits and cost-effectiveness by incorporating user costs, agency costs, and pavement service life into evaluation frameworks, thereby demonstrating the economic feasibility of rollpave technology and supporting large-scale implementation.

## Figures and Tables

**Table 1:** Test results of the performance indexes of special modified asphalt [21,23].

Properties	Results		
	RMB	RMA	Virgin Asphalt
Penetration @ 25 °C (1/10 mm)	63.3	63	67
Ductility @ 25 °C (cm)	88 ^1^	N/A	125
Ductility @ 5 °C (cm)	63	61.8 ^2^	4
Softening point (°C)	At least 100	112	52.4

^1^ Ductility apparatus could not pull it anymore; ^2^ Asphalt demolding occurred when the ductility reached 61.8 cm.

**Table 2:** Types and performance characteristics of bonding coating materials.

References	Paving Methods	Interlayer Bonding Material	Bonding Object	Properties and Characteristics
[46,47,48]	Hot paving	Matrix asphalt	Old and new HMA; HMA and PCC	Bonding performance is better than emulsified asphalt; high energy consumption and difficult to pave.
[49,50]			AC-13 and AC-20	
[51]			AC-20 and C40 PCC	
[51]	Hot paving	Buton Rock Asphalt & SBS modified asphalt	AC-20 and C40 PCC	Bond strength and fatigue life are better than matrix asphalt; high energy consumption and difficult to pave.
[49,50]	Hot paving	SBS modified asphalt	AC-13 and AC-20	Shear fatigue life, shear strength and tensile strength are better than matrix asphalt and SBS modified emulsified asphalt; high energy consumption and difficult to pave.
[52]	Hot paving	Epoxy resin modified asphalt	N/A	Shear strength, tensile strength and drawing strength are lower than epoxy resin; reduced bonding strength in low-temperature and humid environments.
[53]			ESMA-20 and AC	
[54]			N/A	
[49,50]	Hot paving	Epoxy resin	AC-13 and AC-20	Shear fatigue life, shear strength, tensile strength and bending strength are better than SBS modified emulsified asphalt and SBS modified asphalt.
[55]	Hot paving	Polyurethane/epoxy resin modified asphalt	Ultra-high-performance concrete	Excellent tensile properties; high-temperature bonding strength and low-temperature sensitivity; high water resistance and storage stability.
[53]	Cold paving	Emulsified asphalt	ESMA-20 and AC	Bonding performance is lower than epoxy asphalt.
[56]	Cold paving	Super adhesive emulsified asphalt	AC-13 and AC-20	Shear strength and tensile strength are better than emulsified asphalt and SBS modified emulsified asphalt; high water resistance.
[51]	Cold paving	Cationic slow-setting emulsified asphalt	AC-20 and C40 PCC	Strength and fatigue life are lower than matrix asphalt; deformation resistance is better at low temperature; low energy consumption and easy to pave.
[57]			AC-13 and AC-20	
[58]	Cold paving	Cationic slow/fast -setting emulsified asphalt	N/A	Cationic slow-setting emulsified asphalt has greater fracture energy, toughness and ductility than cationic fast-setting emulsified asphalt.
[57]	Cold paving	Fast-break emulsified asphalt	AC-13 and AC-20	Deformation resistance is better at low temperature; bonding strength is greatly affected by temperature.
[49,50]	Cold paving	SBS modified emulsified asphalt	AC-13 and AC-20	Shear fatigue life, shear strength, bending strength and tensile strength are better than matrix asphalt.
[59]	Cold paving	Waterborne epoxy resin emulsified asphalt	SBS-AC-13 and SBS-SC-16	Shear strength and drawing strength are superior to SBS modified asphalt, SBS modified emulsified asphalt, emulsified asphalt and matrix asphalt; good high and low-temperature stability and durability.
[60]			Portland cement concrete slab and AC-10 or UTFL-13	
[61]			AC-20 and C40 cement concrete	
[62]	Cold paving	Waterborne epoxy binders	N/A	High fluidity; adjustable curing speed; no pollution.

**Table 3:** Existing rollpave pavement sections.

Country	Date	Location	Length (m)	Width (m)	Pavement Thickness (mm)	Tracking Result of Pavement Disease ^1^
The Netherlands [5]	November 2001	A50 Apeldoorn, petrol station	100	5	30	-
	August 2002	Delft University of Technology, Lintrack	20	5	30	-
	June 2006	A35 Hengelo, motorway	480	12.5	30	Holes
	November 2006	Deventer, recreational area	30	3	30	-
	January 2007	A37 Nieuw-Amsterdam, motorway	430	11.5	30	Holes
	June 2007	Groningen, industrial area	130	3.5	30	-
	October 2007	A37 Nieuw-Amsterdam, motorway	350	11.5	30	Holes
China [15]	January 2014	Mada Road (X020), Southern Tongzhou District, Beijing	15	1.5	40	Few ruts

^1^ The data comes from visual inspection after 2 years of laying.

**Table 4:** Comparison of several asphalt pavement construction technologies.

Aspect	Conventional Construction	Prefabricated Asphalt Pavement	Rollpave Pavement	Thin Overlay
Material design	Primarily based on conventional asphalt mixtures (e.g., AC, SMA, OGFC), with mix design emphasizing strength and durability	Factory-prefabricated asphalt slabs mainly using conventional asphalt mixtures; slab dimensions must be designed to accommodate transportation and lifting constraints	Factory-produced continuous flexible asphalt mixture strips, emphasizing rollability and interlayer bonding performance; typically requires specially designed polymer-modified asphalt and interlayer bonding agents	Fine-graded or functional thin-layer mixtures (e.g., micro surfacing, ultra-thin wearing courses), with material design focusing on skid resistance, noise reduction, and rapid setting
Performance requirements	Must satisfy structural load-bearing capacity, fatigue life, moisture resistance, and high- and low-temperature performance; overall performance depends on mix design and on-site construction quality	Prefabricated elements require high structural strength, durability, and minimal dimensional deviation; interlayer connection performance is a critical control indicator	Emphasis on adequate flexural performance and high interlayer shear resistance	Mainly aimed at improving surface functional performance (skid resistance, noise reduction, waterproofing), with limited contribution to structural load-bearing capacity
Construction process	Mixing (plant-mixed or in situ) → transportation → paving → compaction → curing	Factory prefabrication → transportation → pavement milling and base preparation → installation→ joint treatment → light compaction → curing	Factory prefabrication into rolls → transportation → pavement milling and base preparation → placement → joint treatment → light compaction	Surface cleaning → application of tack coat → paving → rapid setting
Construction duration	Relatively long construction period, significantly affected by climatic conditions and traffic management	Short on-site construction time; however, the overall duration depends on prefabrication, transportation, and lifting preparation, as well as construction organization and component scale	Compared with conventional asphalt concrete, constructing the full test section with rollpave requires more time, mainly due to the need for a high-quality, smooth binder layer and precise positioning of the mat’s starting point, as well as an immature construction plan. However, unrolling and laying of a single mat is rather fast, averaging 6–10 min for 50–60 m [5,77]	Extremely short construction period; a single carriageway can typically be completed and reopened to traffic within several hours
Economic cost	Mature materials and equipment, relatively low unit cost; however, indirect costs due to long construction duration and traffic delays can be significant	High costs associated with prefabrication, transportation, and lifting equipment, resulting in high initial investment; however, controllable quality and potential advantages in life-cycle cost	High investment in specialized equipment and industrialized production systems, with relatively high direct project costs; nevertheless, extremely short construction time significantly reduces traffic disruption and social costs, offering potential overall economic advantages [11]	Low unit construction cost and simple equipment; mainly suitable for functional improvement with limited structural contribution, typically used as a low-cost maintenance measure
Applicable condition	Suitable for new construction and major rehabilitation projects	Suitable for heavily trafficked roads, time-constrained projects, or special structural sections (e.g., bridge decks and tunnels)	Suitable for rapid repair scenarios on heavily trafficked roads with limited maintenance windows, particularly for maintenance and emergency works	Suitable for functional improvement or preventive maintenance of pavements in good structural condition; not applicable to pavements with severe structural distress

## Data Availability

The original contributions presented in this study are included in the article. Further inquiries can be directed to the corresponding authors.

## Abbreviations

The following abbreviations are used in this manuscript:

SBS	Styrene-Butadiene-Styrene
RMB	Rollpave Modified Bitumen
CERM	Curable Epoxy Resin Mixture
RVD	Ridge-to-Valley Depth
LCCA	Life-Cycle Cost Analysis
